# Microbial nutrient limitations in soil during afforestation in the Mu Us Desert, China: insights from extracellular enzyme stoichiometry

**DOI:** 10.3389/fmicb.2026.1938090

**Published:** 2026-10-21

**Authors:** Shuang Yu, Xiaowei Li, Julian M. Norghauer, Xingdong Wang, Zhongwu Wan, Junlong Yang, Bo Yang, Haotian Yang

**Affiliations:** 1 State Key Laboratory of Ecological Safety and Sustainable Development in Arid Lands, Northwest Institute of Eco-Environment and Resources, Chinese Academy of Sciences, Lanzhou, China; 2 College of Forestry and Prataculture, Ningxia University, Yinchuan, China; 3 Statistical Scientific Editing, Montréal, QC, Canada; 4 Ningxia Lingwu Baijitan National Nature Reserve Administration, Lingwu, China

**Keywords:** afforestation, ecological stoichiometric characteristics, Mu Us Desert, nutrient limitation, soil extracellular enzymes

## Abstract

Afforestation is a key ecological restoration measure for combating desertification in arid and semi-arid regions. But the changes and drivers of nutrient limitations faced by soil microbes during long-term afforestation are unclear. This study investigated soil ecosystems of different-aged stands (planted 10, 30, 50, and 70 years ago) formed by the sand-fixing *Caragana korshinskii* shrub in China’s Mu Us Desert, using extracellular enzyme stoichiometry to infer the dynamics of microbial nutrient limitation. Results showed that establishing *C. korshinskii* sand-fixing vegetation alters the carbon (C), nitrogen (N), phosphorus (P) stoichiometry of soil microbes and their extracellular enzymes. With increasing stand age, soil C: N, C: P, N: P, microbial biomass C: P, and enzyme N: P ratios all increased significantly, peaking at 70 years. However, enzyme C: N peaked much earlier, at 10 years, after which it declined, while enzyme C: P reached its peak at 10 years and then stabilized afterwards. Microbial C- and P- use efficiency both decreased, whereas N-use efficiency initially improved but later weakened. Vector analysis indicated that microbial C limitation intensified during the first decade of shrub stand development (0–10 years), and then stabilized after 10 years, while P limitation declined (0–70 years). Both multiple regression models for VA and VL identified the enzyme C: P as a closely associated variable with microbial nutrient limitations. This work provides scientific insights into how C and N are coupled mechanistically in soil during afforestation of desertland from the perspective of soil–microbe–extracellular enzyme ecological stoichiometry, offering theoretical support for greening desert-like ecosystems and promoting sustainable development in arid and semi-arid regions.

## 1 Introduction

Soil extracellular enzymes mainly originate from soil plants, animals, and microorganisms, and play a pivotal role in the decomposition of soil organic matter and ecosystem cycling of nutrients (Burns et al., 2013). This is because soil extracellular enzymes can convert difficult-to-decompose organic carbon (C), nitrogen (N), and phosphorus (P) polymers belowground into small molecule nutrients directly utilizable by both microorganisms and plants, linking how plants, microorganisms, and soil interact (Liang et al., 2017). In tandem, the activity and chemical stoichiometric characteristics of soil extracellular enzymes are now widely appreciated as reliable tools for evaluating the nutrient and resource acquisition strategies of soil microbiota (Sinsabaugh and Follstad Shah, 2012; Cui et al., 2023). According to resource allocation theory, when the growth of microbes is constrained by the supply of a particular element, they will respond by adjusting their extracellular enzyme secretion strategy, investing more in specific enzymes for obtaining the limiting nutrient, which necessarily alters the ratio of soil enzyme activity (Kunito et al., 2024). Hence, the activity of soil extracellular enzymes and their ecological stoichiometric characteristics can be used to explore and detect the metabolic status of soil microbes and the type of nutrient limitation they incur, in this way serving as effective indicators of soil quality and microbial functioning (Xiao et al., 2023).

With mounting research into ecological stoichiometry and its characteristics, those pertaining to soil extracellular enzymes are gaining more attention. Sinsabaugh et al. (2008) proposed that metabolic and stoichiometric requirements of soil microbes for vital elements such as C, N, and P are reflected in the synthesis of a series of specialized extracellular enzymes. It was later discovered that, on a global scale, the log-transformed activity ratio of soil C-acquisition enzymes (β-1, 4-glucosidase, BG and cellobiohydrolase, CBH), N-acquisition enzymes (β-1, 4-N-acetylglucosaminidase, NAG and leucine aminopeptidase, LAP), and P-acquisition enzymes (alkaline phosphatase, AP) is approximately 1:1:1 (Sinsabaugh et al., 2008). This laid a theoretical foundation for using enzyme activity ratios to assess microbial nutrient limitation. Building on that, Moorhead et al. (2016) introduced an enzyme stoichiometric vector analysis model (‘vector analysis’), to quantitatively describe not only the intensity of C limitation faced by microbes, but also their type and degree of N or P limitation, by calculating the vector length and vector angle. The longer the vector length, the stronger the C limitation, and a vector angle greater than 45° indicates P limitation, while one less than 45° indicates N limitation. Sinsabaugh and Follstad Shah (2012) also established a framework for calculating microbial C-, N-, P-use efficiency values (respectively CUE, NUE, PUE) based on enzyme stoichiometry, providing a fresh perspective for understanding microbial nutrient limitation dynamics through the lens of resource utilization efficiency.

For many arid and semi-arid regions, afforestation offers a promising way to enhance the soil quality of degraded ecosystems now resembling deserts (Yu T. et al., 2024; Wu et al., 2023). Although anticipated changes in soil quality are relatively hard and tedious to observe, the high sensitivity of soil extracellular enzymes’ activity to the changing soil environment means they can serve as informative indicators to assess progress in restoring ecosystems (Wang et al., 2023). There is little doubt that afforestation is capable of improving soil quality, which creates a more favorable belowground habitat for microbial growth and communities, thereby promoting the accumulation of microbial biomass and the secretion of extracellular enzymes (Kaya et al., 2026; Huang et al., 2022). For example, during afforestation of the Loess Plateau, plant community traits and soil properties are closely tied to the nutrient acquisition process of soil microbes, ultimately shaping the activity of soil extracellular enzymes and their ecological stoichiometric characteristics (Zhang et al., 2019). Furthermore, different plant communities will input organic matter with varying properties into soil via their roots, wherein the C, N, and P proportions differ markedly (Wang and Jiao, 2022); this should, in turn, change the availability of nutrients in soil for microbes there. Meanwhile, the microbial community is continuously adjusting its nutritional needs to adapt to an ever-changing soil environment under afforestation. This manifests in subtle-to-stark changes to that community’s structure and further influences the total amount (biomass) of soil microbes, the energy they require for metabolism (C), and the supply/demand dynamics of key nutrient elements (N, P) (Cleveland and Liptzin, 2007). Under divergent soil conditions, microbes may thus encounter disparate levels and distinctive types of energy and nutrient limitations (Cui et al., 2023). To cope with this array of limitations, microbes often increase their secretion of extracellular enzymes to accelerate soil organic matter’s decomposition, enabling them to acquire the ensuing energy and nutrients. It follows that a systematic analysis based on the activity of extracellular enzymes and their C: N: P chemometric relationships is a potentially fundamental tool for determining the nutrient limitation status of soil microbes (Waring et al., 2014). Yet, given the complexity of microbial metabolic limitations in soil, little is known about how they arise during the afforestation process of desert-like ecosystems and to what extent such regularizing mechanism are predictable or generalizable.

In China, the Mu Us Desert is an exemplar for desertification control. Over the past few decades, large-scale artificial sand stabilization and afforestation projects in this region have effectively halted or reversed the extent of land degradation (Xu et al., 2025). The locally dominant xerophytic legume shrub, *Caragana korshinskii*, is widely planted there to construct sand-fixation vegetation stands because of its sought-after traits, namely drought tolerance and nitrogen fixation ability, among others (Zhao et al., 2021). Recent studies have confirmed that planting *C. korshinskii* can considerably increase the soil organic carbon, total nitrogen content (Yu S. et al., 2024), soil microbial C biomass, N content (Zhang et al., 2020), and the activity of various enzymes, such as sucrase, urease, and alkaline phosphatase (Li et al., 2021). Nevertheless, in a broader context, most research to date tends to focus on single-factor changes in the nutrient content, microbial biomass, or enzyme activity in soils during restoration via greening. Moreover, long-term afforestation outcomes over a 70-year horizon remain unclear. In particular, the complementary shifts in soil–microbe–extracellular enzyme chemical composition and dynamic changes to microbial nutrient limitation are still unknown, as are their underlying mechanistic drivers. This knowledge gap precludes a richer understanding of the full potential of restoration using sand-fixing woody plants, especially legume species (Fabaceae). This study examined *C. korshinskii* stands differing in age (10, 30, 50, and 70 years since first planted) in the Mu Us Desert, to study how vegetation restoration impacts microbial nutrient limitation in soil. We aimed to (1) clarify the evolving patterns of soil–microbe–extracellular enzyme chemical composition across restoration stages (years of stand age); (2) determine trends in the type and strength of soil microbial nutrient limitation; (3) explore the major drivers (factors) affecting microbial nutrient limitation dynamics. The findings provide a scientific basis for an in-depth understanding of microbial nutrient regulation mechanisms in the artificial afforestation process of desertland and provide timely theoretical support for adaptive management of ecological restoration in arid areas.

## 2 Materials and methods

### 2.1 Research sites

The study area sits in the southwestern edge of the Mu Us Desert, within the Baijiatan Nature Reserve of Lingwu City, Ningxia at 37°54’–38°22’N, 106°23’–106°48’E (Figure 1). The area’s average elevation is 1,288 m, and it has a temperate continental semi-arid monsoon climate with an annual average (i) precipitation of 192.90 mm, concentrated from June to September, (ii) potential evaporation of 1933.3 mm, and (iii) temperature of 8.8 °C. Sandy soil is most predominant here, followed by gray calcareous and saline-alkali gray calcareous soil types. Accordingly, sandy vegetation is widespread, consisting mainly of *Artemisia desertorum*, *Agriophyllum pungens*, *Hedysarum scoparium*, *C. korshinskii*, and *Salix psammophila*, whose plant community coverage ranges from 2 to 20% (Zhan and Han, 2015). Of these, *A. desertorum* and *C. korshinskii* are dominant shrubs, playing a critical ecological role by stabilizing desert sand in this area. A ground view of the research sites is shown in Figure 2. Prior to undergoing ecological restoration, this study area had typical mobile dune landform features, with severe wind erosion and virtually no surface vegetation coverage.

**Figure 1:**
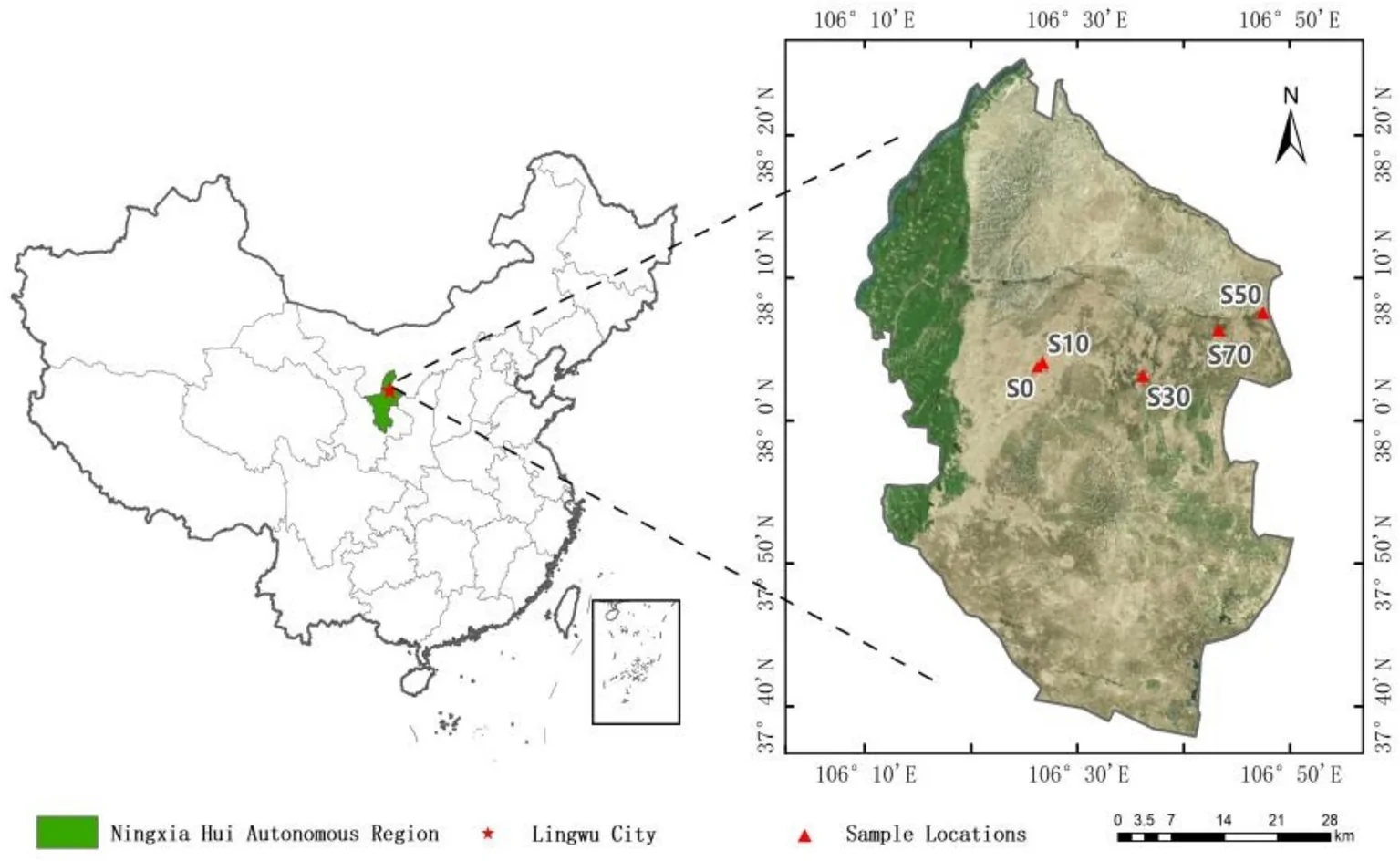
The study area in northern China and its sampling sites corresponding to four extensive *Caragana korshinskii* shrub stands differing in age: 10 years (S10), 30 years (S30), 50 years (S50), 70 years old (S70). The fifth site was the control, a mobile sand dune (S0) where no afforestation occurred.

**Figure 2:**
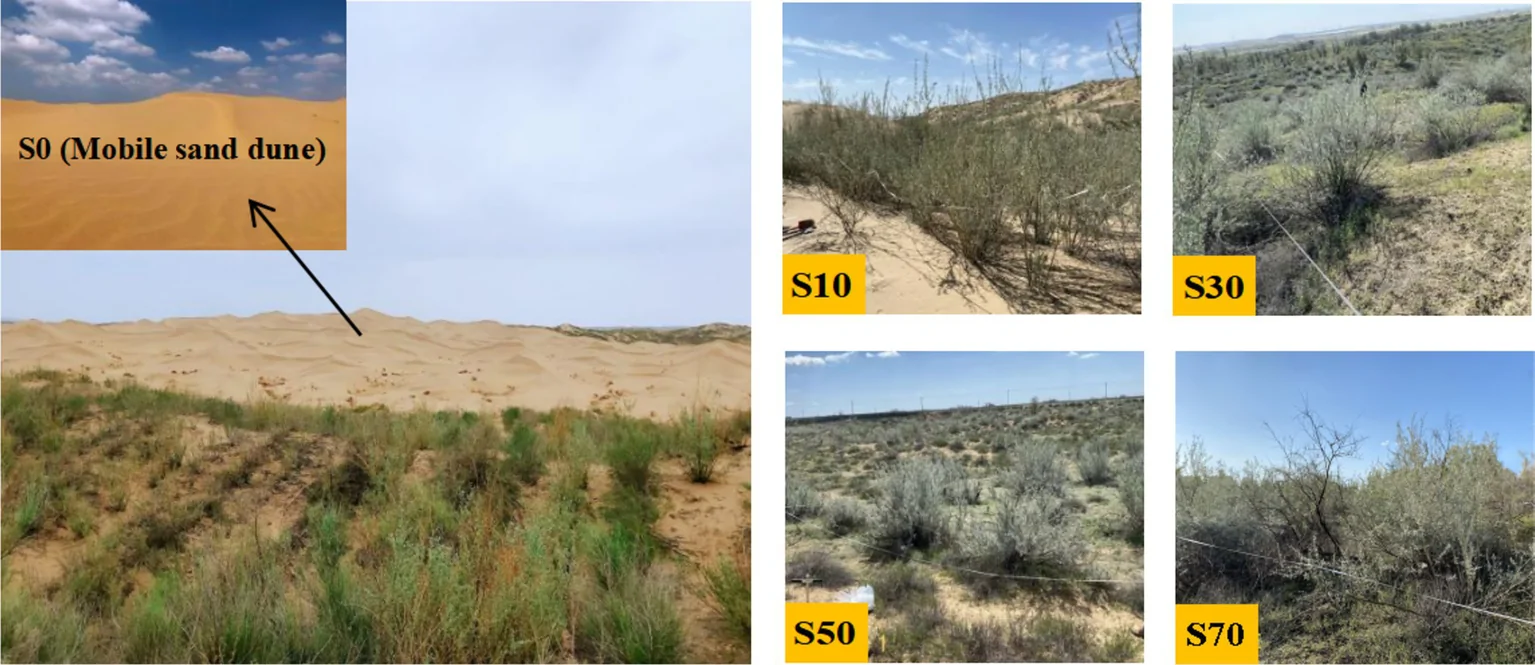
General view of the five sampling sites. The bare dune in upper part of the far-left panel exemplifies the initial state.

### 2.2 Experimental design

Because the different-aged *C. korshinskii* stands (i.e., differing in years since establishment) had the same initial pre-planting conditions in terms of soil type, seedlings, and planting density. Early in August 2021, using a space-for-time substitution approach, we selected four different-aged (10, 30, 50, and 70 years old) plantations of *C. korshinskii* to form a chronosequence of stands corresponding to a restoration gradient. At each afforestation site and the mobile sand dune (control site), we set three replicate sampling plots (each 10 × 10 m)—similar in terrain and local climate, soil parent material, and historical land-use conditions—at least 100 m apart, to ensure their independence while mitigating the influence of within-site spatial heterogeneity. Hence, a total of N = 15 sampling plots were set up—their altitude and longitude/latitude recorded—from which soil was collected.

### 2.3 Collection and analysis of soil samples

Soil samples were collected in August (of 2021), when plants tend to grow most vigorously and microbial action is peaking. In each 100-m^2^ plot, soil samples from the 0–10 cm depth layer were obtained, using a 5-cm diameter soil auger and the five-point sampling method, then thoroughly mixed to yield a single composite sample per plot. To remove roots and debris, these composite soil samples (n = 15) were passed through a 2-mm diameter sterile sieve, and then split and transferred into sterile bags as follows: One part of each soil sample was immediately placed in a constant low-temperature (4 °C) box for cold chain transportation to the laboratory, while the other part was air-dried.

To determine the soil organic carbon (SOC) content, the external heating method with potassium dichromate was used; for soil total nitrogen (STN) content, a BUCHI-K360 automatic Kjeldahl nitrogen analyzer was used; for soil total phosphorus (STP) content, a Skalar continuous-flow analyzer was used. These three soil variables were quantified by following the protocols outlined in Bao (2000). The microbial biomass carbon (MBC), microbial biomass nitrogen (MBN), and microbial biomass phosphorus (MBP) measurements were made using the chloroform fumigation extraction method (Wu et al., 2006). To determine the respective activity of β-1, 4-glucosidase (BG), cellobiohydrolase (CBH), β-1, 4-N-acetylglucosaminidase (NAG), leucine aminopeptidase (LAP), and alkaline phosphotase (AP), we employed the microplate fluorescence method as described by Saiya-Cork et al. (2002). Soil enzyme activities were assayed by the microplate fluorescence method with appropriate buffers (sodium acetate buffer [pH 5.0] for BG, CBH, NAG; sodium bicarbonate buffer [pH 11.0] for AP; and sodium borate buffer [pH 8.5] for LAP). Substrate concentrations were 200 μmol·L^−1^ for all assays. Samples were incubated at 25 °C for 4 h, after which their respective fluorescence was measured at 365 nm excitation and 450 nm emission. All enzyme activities are expressed in nmol·g^−1^·h^−1^.

Next, three soil stoichiometric ratios were calculated on a mass basis, as follows: soil carbon-to-nitrogen ratio (soil C: N) = SOC/STN; soil carbon-to-phosphorus ratio (soil C: P) = SOC/STP; soil nitrogen-to-phosphorus ratio (soil N: P) = STN/STP. Likewise, stoichiometric ratios in soil microbial biomass were also calculated, as follows: microbial biomass carbon-to-nitrogen ratio (MB C: N) = MBC/MBN; microbial biomass carbon-to-phosphorus ratio (MB C: P) = MBC/MBP; microbial biomass nitrogen-to-phosphorus ratio (MB N: P) = MBN/MBP.

### 2.4 Ecological stoichiometry of soil extracellular enzymes and microbial nutrient limitations

This study used three ratios in the activity of specific extracellular enzymes in soil to characterize (and infer) the resource allocation strategies of microorganisms with respect to carbon, nitrogen, and phosphorus nutrients. Their calculation is based on the standardized model proposed by Sinsabaugh et al. (2009), as follows:


$${\mathrm{EEA}}_{C:N}=\frac{\ln(\mathrm{BG}+\mathrm{CBH})}{\ln(\mathrm{NAG}+\mathrm{LAP})}$$

$${\mathrm{EEA}}_{C:P}=\frac{\ln(\mathrm{BG}+\mathrm{CBH})}{\ln(\mathrm{AP})}$$

$${\mathrm{EEA}}_{N:P}=\frac{\ln(\mathrm{NAG}+\mathrm{LAP})}{\ln(\mathrm{AP})}$$

Where *EEA* denotes the soil extracellular enzyme activity; the term *BG+CBH* represents the soil microbial C acquisition enzymes; *NAG+LAP* represents the soil microbial N acquisition enzymes; and *AP* represents the soil microbial P acquisition enzyme (Sinsabaugh et al., 2009; Waring et al., 2014). In all cases, the numerator and/or denominator are the sums of the respective enzyme activities after their natural-log transformation.

To quantitatively analyze the characteristics of soil microbial metabolic limitations, we applied the enzyme quantification vector model (Moorhead et al., 2013, 2016). This model, which is based on the ratio of extracellular enzyme activities of microorganisms, can be used to convey the types and intensities of nutrient limitations in a two-dimensional vector space. Its calculations are as follows:

$$\text{Vector length}=\text{SQRT}\;(x^{2}+y^{2})$$

Vector angle(°)=DEGREES(ATAN2(x,y))

Where *x* is the relative enzymatic activity of C vis-à-vis P, that is, *x* = (BG + CBH): AP; and, *y* is the relative enzymatic activity of C vis-à-vis N, that is, *y* = (BG + CBH):(NAG+LAP). The vector length reflects the relative C limitation faced by soil microbes, with a longer length indicating a greater C limitation, whereas the vector angle conveys the N or P limitation faced by soil microbes. An angle of 45°–90° indicates P limitation, for which a larger angle means stronger P limitation. Conversely, an angle of 0°–45° is interpreted as N limitation, for which a smaller angle means stronger N limitation (Moorhead et al., 2013, 2016).

The microbial CUE (C-use efficiency) is derived this way (Sinsabaugh and Follstad Shah, 2012):

$$\mathrm{CUE}=\mathrm{CU}E_{\max}\times {\left\{\frac{S_{C:N}\times S_{C:P}}{\left(K_{C:N}+S_{C:N})\times (K_{C:P}+S_{C:P}\right)}\right\}}^{0.5}$$

$$S_{C:N}=\frac{B_{C:N}}{L_{C:N}}\times \frac{1}{\mathrm{EE}A_{C:N}}$$

$$S_{C:P}=\frac{B_{C:P}}{L_{C:P}}\times \mathrm{EE}A_{C:P}$$

Where *L_C: N_* is the soil carbon-nitrogen ratio; *L_C: P_* is the soil carbon-phosphorus ratio; *B_C: N_* is the soil microbial biomass carbon-nitrogen ratio; *B_C: P_* is the soil microbial biomass carbon-phosphorus ratio; *K_C: N_* is the half-saturation constant 0.5; *K_C: P_* is the half-saturation constant, 0.5; and *CUE_max_* is fixed at 0.6 (Wu et al., 2019).

The calculation formulas for N-use efficiency (NUE) and P-use efficiency (PUE) of microbes are (Sinsabaugh and Follstad Shah, 2012):

$$\mathrm{NUE}=\mathrm{NU}E_{\max}\times \frac{S_{N:C}}{S_{N:C}+K_{N:C}}$$

$$S_{N:C}=(1-\mathrm{EE}A_{N:C})\frac{B_{N:C}}{L_{N:C}}$$

$$\mathrm{PUE}=\mathrm{PU}E_{\max}\times \frac{S_{P:C}}{S_{P:C}+K_{P:C}}$$

$$S_{P:C}=(1-\mathrm{EE}A_{P:C})\frac{B_{P:C}}{L_{P:C}}$$

Where *EEA_N: C_* is (NAG+LAP):(BG + CBH); *L_N: C_* is the soil nitrogen-carbon ratio; *B_N: C_* is the soil microbial biomass nitrogen-carbon ratio; *K_N: C_* is the half-saturation constant 0.5; *NUE_max_* is 1.0; *EEA_P: C_* is AP:(BG + CBH); *L_P: C_* is the soil phosphorus-carbon ratio; *B_P: C_* is the soil microbial biomass phosphorus-carbon ratio; *K_P: C_* is the half-saturation constant 0.5; and *PUE_max_* is fixed at 1.0 (Wu et al., 2019).

### 2.5 Data analysis

The effects of different stand ages on soil physicochemical properties and chemical stoichiometry, soil microbial biomass and chemical stoichiometry, extracellular enzyme activity and chemical stoichiometry were analyzed, using SPSS 20.0 software, by univariate ANOVAs (one-way analysis of variance) followed by least significant difference tests (for the pairwise comparison of means). Graphs were drawn in Origin 2021 software.

Next, with a view to parsimonious prediction, we wanted to distinguish major factors influencing VA and VL, the two kinds of microbial nutrient limitation. We first conducted an redundancy analysis (RDA) of the data matrix (15 plots with 2 response variables + 12 explanatory variables: soil, microbial, extracellular enzyme stoichiometric characteristics and nutrient utilization efficiency). However, with just N = 15 plots for the entire chronosequence, the ratio of sample size to all Y, X variables precluded any sound statistical inference. So, to minimize that, it was simpler to fit two multiple linear regression models (MLRs), one for the VA and another for VL, using a stepwise procedure (forward selection, with backward elimination; Armstrong and Hilton, 2010) under default settings in Genstat software (18th ed.). This approach is reasonable, given that RDA is a logical extension of MLR. Both final obtained MLRs (reduced to three or four predictor terms) were checked for model assumptions of normality of residuals and homogenous variance by examining plots of their standardized residuals (histograms, fitted values, normal probability, and half-normal graphs). Due to strong collinearity between EEA C: P and EEA N: P (Pearson’s r = 0.84, *p* < 0.001), the latter was omitted (and vice-versa), in this modeling.

## 3 Results

### 3.1 Changes in soil and microbial nutrients

The SOC, STN, and STP contents differed among the 10- to 70-year-old shrub stands and the control (mobile sand dune) (Table 1, all *p* < 0.05). All three soil nutrients increased with longer afforestation in place, being greatest after 70 years at 14.08, 0.51 and 0.15 g·kg^−1^, respectively. The patterns for mean soil C: N, soil C: P and Soil N: P were basically the same, in tending to increase over time, whose ratios at the 70-year mark significantly surpassed those in the control dune and younger shrub stands (Tables 1, *p* < 0.05).

**Table 1:** Changes over time in the chemical composition of soil and microbes, and their respective ratios during the afforestation chronosequence of the Mu Us Desert in China.

Variable	Age of the shrub (*Caragana korshinskii*) stands				
	0 years	10 years	30 years	50 years	70 years
SOC (g·kg^−1^)	1.43 ± 0.05 e	2.76 ± 0.15 d	4.16 ± 0.23 c	8.08 ± 0.19 b	14.08 ± 0.62 a
STN (g·kg^−1^)	0.08 ± 0.01 e	0.16 ± 0.01 d	0.20 ± 0.02 c	0.32 ± 0.01 b	0.51 ± 0.01 a
STP (g·kg^−1^)	0.131 ± 0.001 d	0.133 ± 0.001 d	0.140 ± 0.012 c	0.145 ± 0.001 b	0.15 ± 0.03 a
Soil C: N	17.58 ± 0.34 c	17.59 ± 0.35 c	20.50 ± 1.11 b	25.67 ± 1.49 a	27.58 ± 1.66 a
Soil C: P	10.864 ± 0.48 e	20.74 ± 1.33 d	29.63 ± 1.81 c	55.84 ± 1.49 b	91.69 ± 5.03 a
Soil N: P	0.62 ± 0.04 e	1.18 ± 0.08 d	1.45 ± 0.15 c	2.17 ± 0.07 b	3.32 ± 0.12 a
MBC (mg·kg^−1^)	7.86 ± 0.09 e	13.81 ± 2.09 d	23.643 ± 0.73 c	40.00 ± 1.39 b	72.80 ± 0.45 a
MBN (mg·kg^−1^)	1.99 ± 0.16 d	3.11 ± 0.09 d	5.58 ± 0.32 c	7.19 ± 0.01 b	12.53 ± 1.41 a
MBP (mg·kg^−1^)	6.88 ± 0.28 e	8.46 ± 0.26 d	15.71 ± 0.63 c	23.88 ± 0.66 b	34.13 ± 0.75a
MB C: N	3.97 ± 0.28 b	4.43 ± 0.53 b	4.24 ± 0.26 b	5.57 ± 0.19 a	5.85 ± 0.66 a
MB C: P	1.15 ± 0.06 c	1.63 ± 0.26 b	1.51 ± 0.02 b	1.68 ± 0.06 b	2.13 ± 0.03 a
MB N: P	0.29 ± 0.02 b	0.37 ± 0.02 a	0.36 ± 0.02 a	0.30 ± 0.01 b	0.37 ± 0.04 a

SOC, soil organic carbon; STN, soil total nitrogen; STP, soil total phosphorus; soil C: N, SOC/STN; soil C: P, SOC/STP; soil N: P, STN/STP; MBC, microbial biomass carbon; MBN, microbial biomass nitrogen; MBP, microbial biomass phosphorus; MB C: N, MBC/MBN; MB C: P, MBC/MBP; MB N: P, MBN/MBP. Values are the mean ± standard error, *n* = 3 plots. Different letters within the same row indicate significant differences (*p* < 0.05) among different-aged stands.

Likewise, MBC, MBN, and MBP all increased across the sampled afforestation chronosequence and reached their highest values at 70 years (Table 1, all *p* < 0.05). The MB C: N ratio fluctuated with longer years since planting, but was also greatest at 70 years, followed by 50 years; both significantly surpassed that in the 10- and 30-year-old stands and the control dune whose values were similar (Table 1). The MB C: P ratio also reached its maximum at 70 years, being significantly higher than in any younger shrub stand, including the control dune (Tables 1, *p* < 0.05); while similar among stands aged 10, 30, and 50 years (*p* > 0.05), their MB C: P ratios were significantly higher than the control dune’s (*p* < 0.05). In contrast, the MB N: P ratio was similarly greatest at 10, 30, and 70 years, being significantly higher than in the 50-year-old stand or the control dune (Tables 1, *p* < 0.05).

### 3.2 Ecological stoichiometric changes of extracellular enzymes in soil

Results for extracellular enzyme activity in the soil and their chemical ratios are shown in Figure 3. All acquisition enzymes for carbon (BG + CBH), nitrogen (NAG+LAP), and phosphorus (AP) from soil increased through the afforestation chronosequence, being most active after 70 years, when they significantly exceeded those in younger shrub stands, as well as the control dune (Figures 3a–c, all *p* < 0.05). After planting *C. korshinskii*, the EEA C: N ratio initially increased, reaching its significantly highest value at 10 years, after which it declined (Figure 3d; *p*<0.05). The EEA C: P ratio also rose early and peaked in the 10-year-old stand, but its differences vis-à-vis the older shrub stands were marginal, albeit statistically significant (Figure 3e; *p*< 0.05). In contrast, the EEA N: P ratio steadily increased as each stand got older, in a significant step-wise manner (Figure 3f; *p*< 0.05).

**Figure 3:**
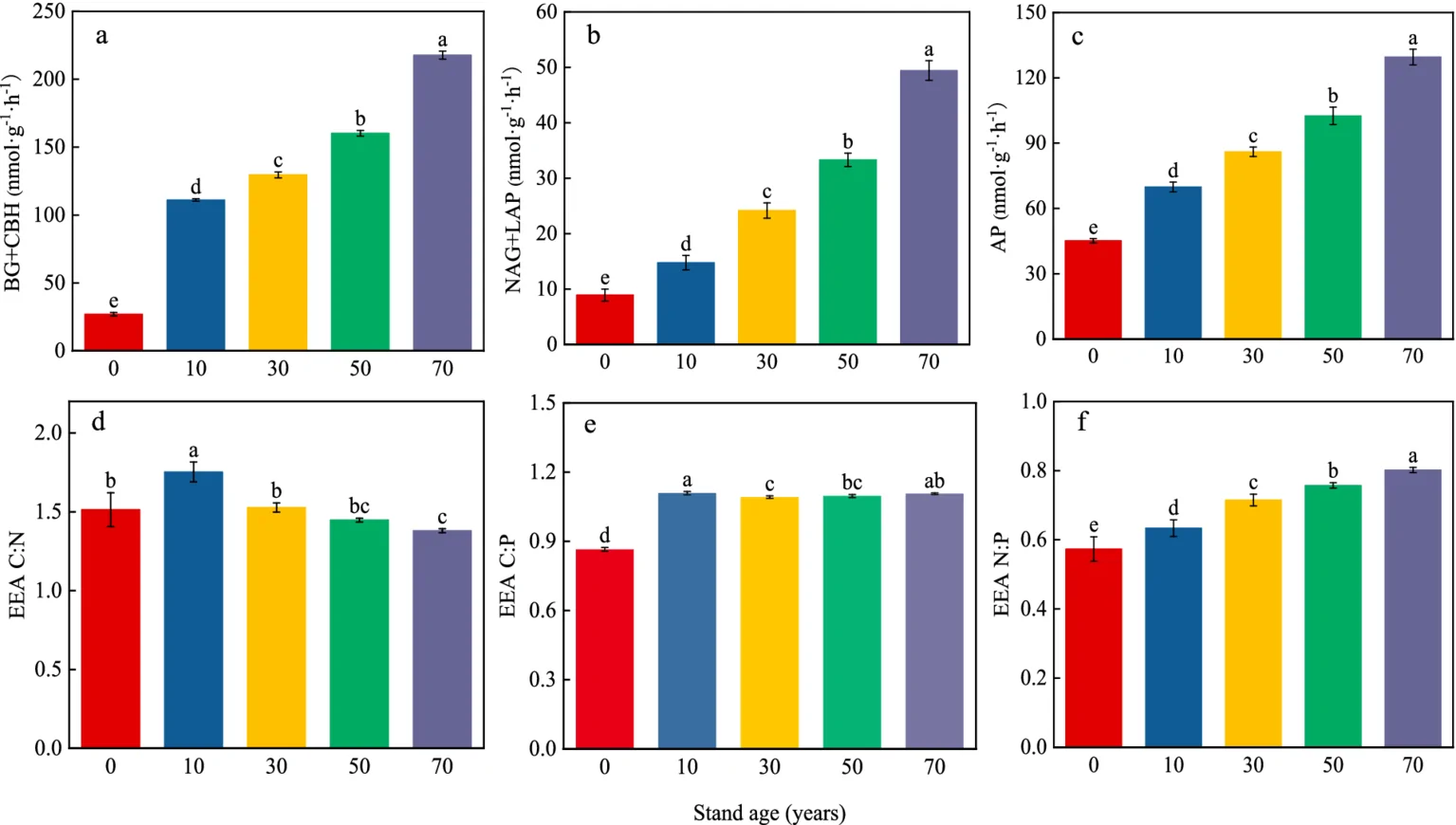
Changes in the extracellular enzymes and their chemical composition characteristics in soils of different forest ages. **(a)** BBG + CBH, carbon-acquisition enzyme (β-1, 4-glucosidase, cellobiohydrolase); **(b)** NAG+LAP, nitrogen-acquisition enzyme (β-1, 4-N-acetylglucosaminidase, leucine aminopeptidase); **(c)** AP, phosphorus-acquisition enzyme (alkaline phosphatase); **(d)** EEA C:N; **(e)** EEA C:P; **(f)** EEA N:P. EEA, extracellular enzyme activity. Bars are the mean ± standard error, *n* = 3 plots. Different letters indicate significant differences at *p* < 0.05 among different-aged stands of the sand-fixing shrub (*Caragana korshinskii*) in the Mu Us Desert.

### 3.3 Soil microbial utilization efficiency

Among stages in the afforestation chronosequence, their ranking for mean microbial C-use efficiency was 0 > 70 > 10 > 50 > 30 years. Microbial C-use efficiency was significantly higher in soil of the control (mobile sand dune) than that of any subsequent stage (Figure 4a; *p*< 0.05). The microbial N-use efficiency of soil increased substantially in the first 10 year of afforestation, after which it tended to weaken, though its value at 70 years was still significantly greater than in the control dune (Figure 4b). For microbial P-use efficiency, however, it ranked only slightly differently across the chronosquence, as follows: 0 > 10 > 50 > 70 > 30 years (Figure 4c; *p*< 0.05).

**Figure 4:**
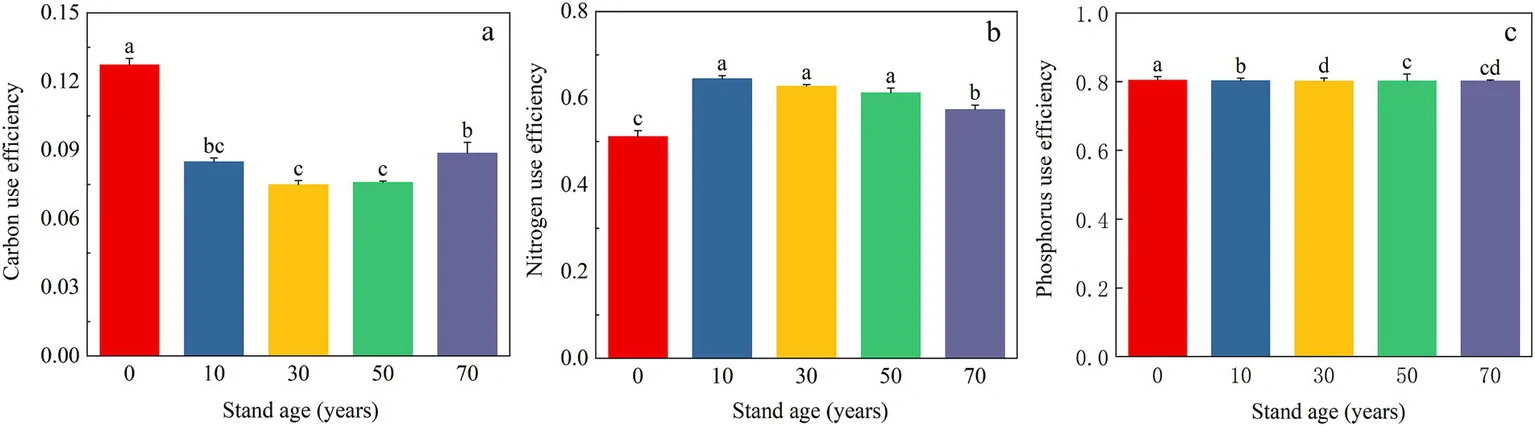
Changes in the **(a)** CUE, **(b)** NUE, and **(c)** PUE of soil in different stages of the afforestation chronosequence. Bars are the mean ± standard error, *n* = 3 plots. Different letters indicate significant differences at *p* < 0.05 among different-aged stands of the sand-fixing shrub (*Caragana korshinskii*) in the Mu Us Desert.

### 3.4 Nutrient limitations of soil microbes

As Figure 5a shows, ratios for the quantified soil extracellular enzymes quantification shift with the duration of afforestation, deviating substantially from control dune once shrubs were planted. This suggests that the greening (ecological restoration) of the Mu Us Desert influenced how soil microbes acquire nutrients. Fitting the enzyme quantification vector model revealed that microbial C limitation varied significantly across the chronosequence (*p* < 0.05), in this order: 10 > 50 > 70 > 30 > 0 years since planting (Figure 5c). According to this vector analysis of the enzyme activity quantification ratio, a greater distance from a given data point (sampled plot) to the origin represents a greater intensity of microbial C limitation. When a point lies to the upper-left area of the 1:1 diagonal line, it indicates that soil microbes are mainly limited by P; but if situated instead to its lower-right area, this implies they are mainly limited by N. And if the point lies on the diagonal, or a group straddles it, this would indicate the soil microbial community faces a dual limitation of N and P. Here, all replicate plots spanning the chronosequence are located above the 1:1 diagonal line, suggesting that soil microbes are mainly P-limited during the greening of desertland (Figure 5a). Specifically, among the different-aged shrub stands and the control dune, the temporal change in microbial P limitation is significant (*p* < 0.05), taking this order: 0 > 10 > 30 > 50 > 70 years. Notably, microbes were most P-limited in the absence of vegetation (control dune = 0 years), being also significantly more P-limited at 10 and 30 years than at either 50 or 70 years of afforestation (Figure 5d). Further, there is an inverse relationship between the relative microbial C and P limitations (*p* < 0.01): as the former increases the latter limitation decreases (Figure 5b).

**Figure 5:**
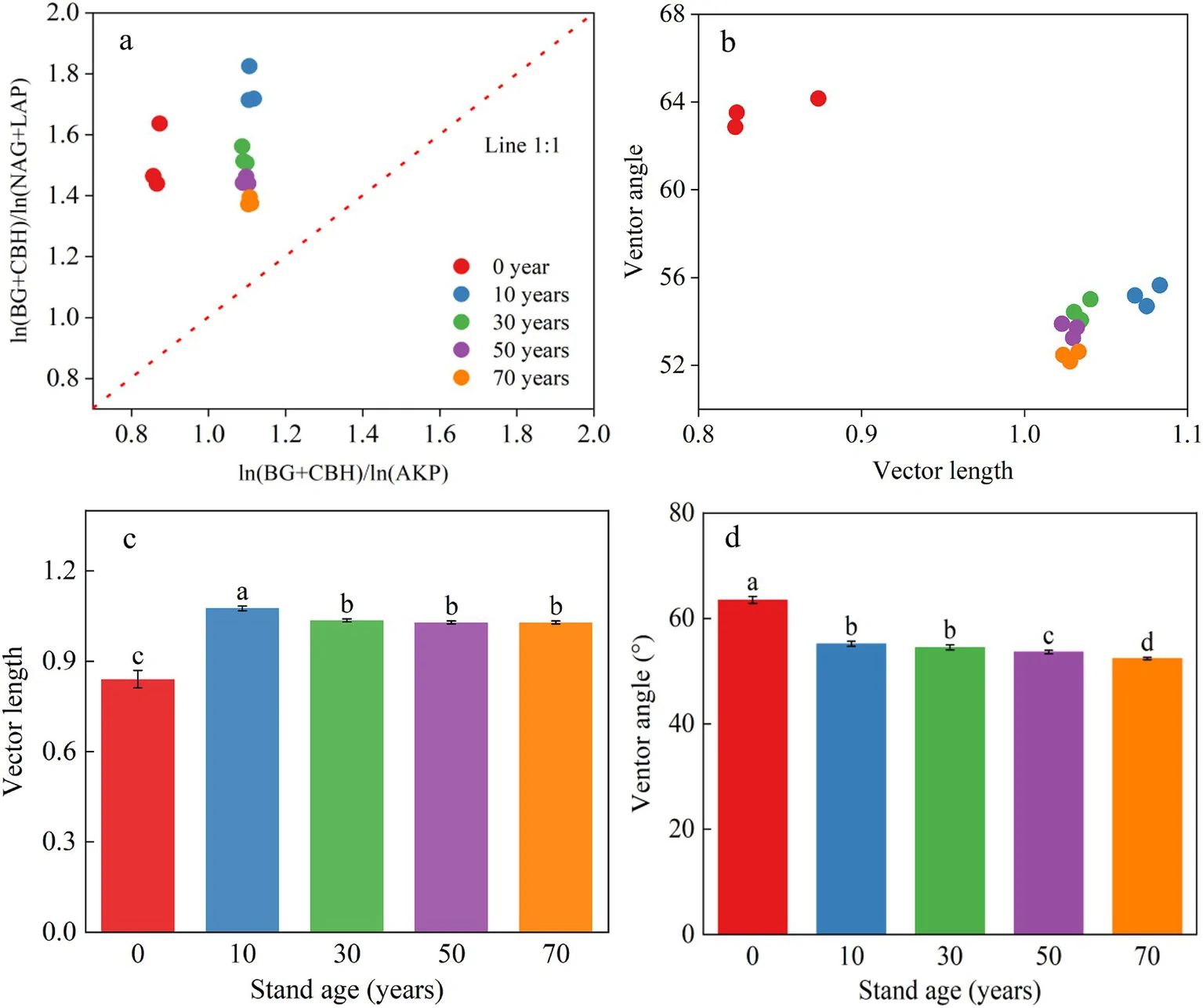
Scatterplot of ln(BG+CBH)/ln(NAG+LAP) versus ln(BG+CBH)/ln(AP) showing N vs P limitation areas **(a)**. Vector angle (VA) versus vector length (VL) by stand age **(b)**. Relative nutrient limitations of soil microbes during afforestation and their relationship. In panels **(c)** and **(d)**, bars are the mean ± standard error, *n* = 3 plots. Different letters indicate significant differences at *p* < 0.05 among different-aged stands of the sand-fixing shrub (Caragana korshinskii) in the Mu Us Desert.

### 3.5 Factors associated with the nutrient limitations faced by soil microbes during afforestation

We used MLR (multiple linear regression) to examine how microbial nutrient limitation, expressed as VA or VL, was associated with soil physicochemical factors and their chemical composition characteristics in the different-aged afforestation stands (including the dune control). Virtually all the variation in the VA response (> 99%, Mallows Cp = 4.0) could be explained by retaining EEA C: P, EEA C: N, and NUE as significant predictors (MLR, *F*_3, 11_ = 8,258, *p* < 0.001; Table 2 top). However, closer inspection revealed that EEA C: P was clearly the most meaningful factor, given its more than 40-fold higher partial regression coefficient vis-à-vis EEA C: N, and its already very strong negative linear relationship to VA (Figure 6a). For VL, both EEA C: P and EEA C: N also emerged as closely associated variables, supplemented marginally by PUE and Soil C: N (MLR, *F*_4, 10_ = 2,466, *p* < 0.001; Table 2 bottom). But again, EEA C: P was by far the most strongly associated variable involved, owing to its pre-existing tight, though now positive, relationship to this form of microbial nutrient limitation (Figure 6b). Rerunning both MLRs with EEA N: P substituted for EEA C: P gave non-satisfactory results (unequal variance, poor residual normality), in part because of nonlinearity between EEA N: P and VA, VL.

**Table 2:** Results for each stepwise multiple linear regression model (MLR) for microbial nutrient limitation (VA: vector angle, VL: vector length) as a function of soil- or microbe-related nutrient factors (*N* = 15 plots spanning the afforestation chronosequence).

Response	Parameter	Coefficient	S. E.	*t* value	*p*-value
VA	Constant	90.82	0.478	189.94	
	EEA C: P	−42.09	0.462	−91.19	< 0.001
	EEA C: N	4.02	0.350	11.48	< 0.001
	NUE	4.55	1.18	3.86	0.003
VL*	Constant	7.92	1.44	5.51	
	EEA C: P	0.6245	0.0200	31.16	< 0.001
	EEA C: N	0.1602	0.00843	19.00	< 0.001
	PUE	−9.79	1.78	−5.52	< 0.001
	Soil C: N	0.001145	0.000214	5.34	< 0.001

EEA, extracellular enzyme activity in soil; PUE, phosphorus-use efficiency; NUE, nitrogen-use efficiency; * In the stepwise procedure for this respinse, model checking uncovered gross non-linearity in a formerly significant explanatory variable, MB C: N (*p* = 0.034), so it was excluded; likewise for a non-significant MB C: P; after refitting, both NUE and Soil N: *p* were non-significant (*p* > 0.01 and *p* > 0.02, respectively) and thus dropped, leaving a four-term MLR.

**Figure 6:**
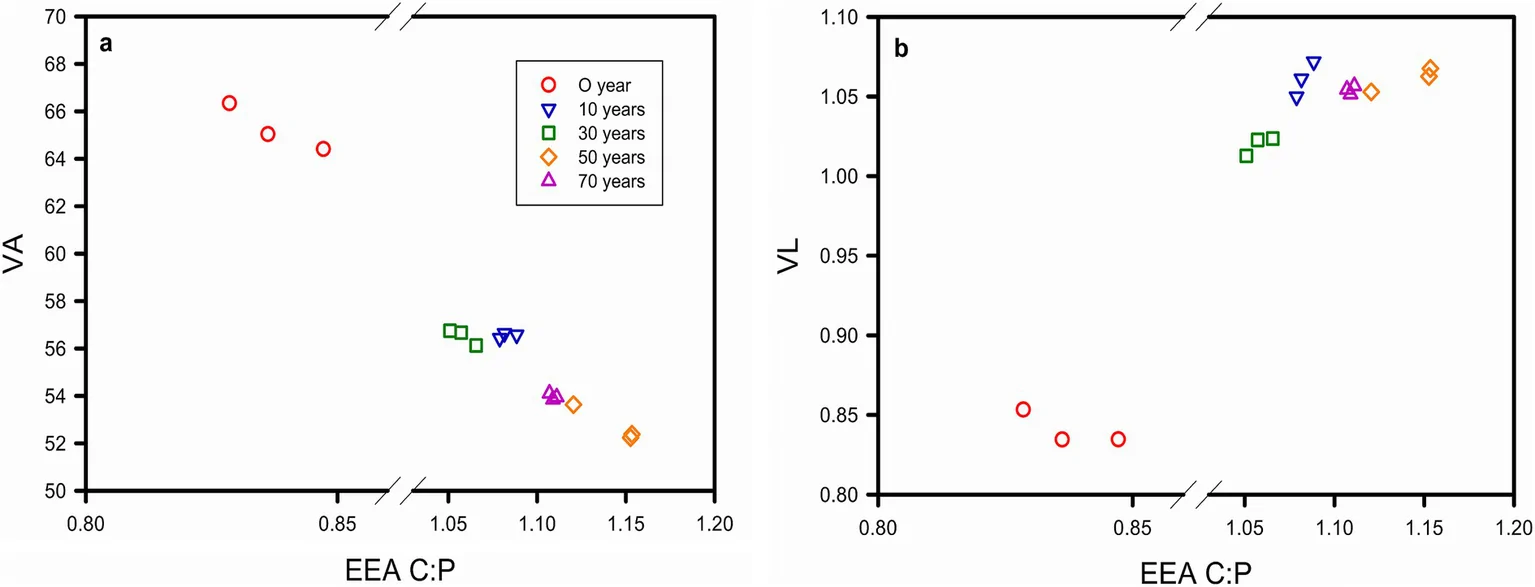
Relationship of extracellular enzyme activity (EEA) in soil for C: P to microbial nutrient limitation expressed as vector angle (VA) **(a)** and vector length (VL) **(b)** (see Methods).

## 4 Discussion

### 4.1 Chemical stoichiometric characteristics of soil-microbe extracellular enzymes after planting *C. korshinskii* shrubs in the mu Us Desert

The soil C: N: P ratio indicates the cycling of C, N, and P elements, but also reflects the content of soil organic matter and the nutrient balance status. This study found that during afforestation of the Mu Us Desert, the values of soil C: N, soil C: P, and soil N: P all increased over time. This is consistent with findings by Zhang et al. (2016) for different-aged *Haloxylon ammodendron* forests planted in desert oasis areas. The increase in soil C: N is particularly consistent with SOC accumulating faster than STN, indicating that plant-derived carbon inputs increasingly contributed to the soil organic matter pool as *C. korshinskii* stands developed. Repeated litter inputs, root-derived carbon, and microbial processing of these substrates likely strengthened the formation and retention of soil organic matter, while N fixation by this leguminous shrub provided an additional pathway for N accumulation. Thus, the increasing soil C: N and N: P ratios suggest that afforestation progressively changed the balance between carbon inputs, N supply, and P availability rather than simply increasing all nutrients at the same rate (Cleveland and Liptzin, 2007; Jia et al., 2022; Huang et al., 2025).

The soil C: P ratio, a critical indicator of phosphorus exchange capacity between soil organic matter and the environment, also increased substantially, reaching 91.69 at 70 years—well above China’s national average of 60.0 (Tian et al., 2010). When soil C: P exceeds ~200, microbial C increases rapidly and P undergoes net fixation; conversely, below 200, P undergoes net mineralization (Arenberg and Arai, 2019). All stands in this study fell below that threshold, but the upward trajectory suggests that, as stands developed, the rate of soil organic matter decomposition and the relative availability of P declined in tandem. The upward temporal trend in the soil N: P ratio is in line with soil changes reported during land restoration using conifer plantations of *Pinus sylvestris* (Shu et al., 2018). This similar phenomenon could be due to the gradual diversification of soil N-acquisition mechanisms. With more litter input undergoing decomposition, soil N accumulates, leading to a continuous build-up of soil N as its stands develop over decades.

The C: N: P chemical stoichiometric characteristics of soil microbial biomass are recognized as key mechanisms regulating the life-history strategies of microorganisms and governing whether nutrients are released during litter decomposition (Heuck et al., 2015); they also regulate the availability of soil nutrients. In our study, the per plot ratios of MB C: N, MB C: P and MB N: P were 3.67–7.35, 1.19–2.84, and 0.24–0.38, respectively. These values are notably lower than their corresponding global averages (7.6, 42.40, 5.60; Cleveland and Liptzin, 2007). This is especially so for MB C: P, given the low content of MBC and MBP in sandy soil. The low MB C:*p* values are biologically plausible, as sandy desert soils typically have limited phosphorus availability, which constrains microbial biomass P accumulation relative to C. Extracellular enzyme activity (EEA) is now widely used to gage the energy acquisition and nutrient utilization status of microorganisms during their life cycle, and this activity is closely coupled with the geochemical cycling process of essential elements such as C, N, and P (Adamczyk et al., 2014). The chemical stoichiometric ratio of EEA is a useful indicator for distinguishing the intensity of soil microbes’ demand for C, N, P and other elements, and it also reflects the balance of soil nutrient supply (Schimel and Weintraub, 2003). Our study shows that, along the afforestation chronosequence in the Mu Us Desert, the EEA C: N undergoes a ‘rise then fall’ process, diminishing considerably after 10 years (*p* < 0.05). This suggests that microbes reduced their investment in secreting enzymes related to C source acquisition, implying that the limitation of C resources they face weakens over time. Meanwhile, the EEA C: P ratio peaked at 10 years and then stabilized, whereas the EEA N: P ratio continued to increase with longer afforestation periods, indicating a greater relative demand for P by of soil microbes as the shrub stands age.

### 4.2 Soil microbial nutrient-use efficiency and constraining factors during afforestation of the mu Us Desert with the shrub (*C. korshinskii*)

The enzymatic stoichiometric vector angle, as a quantitative indicator for measuring the intensity of microbial nutrient limitation, is a powerful tool for inferring the relative limiting states of C, N, and P. In this study, across the entire 0–70-year afforestation chronosequence in the Mu Us Desert, the external enzyme vector angle always stayed above 45°, indicating that soil microbes in this region are chronically P-limited. The most likely explanation is that, with continual stand development, an improving canopy structure promotes the colonization by herbaceous plants and their growth (Guo et al., 2025). The augmented diversity of these understory herbs and overall greater aboveground plant biomass together may intensify a burgeoning demand for P by vegetation, thus weakening the opportunities for soil microbes to obtain P (Jiao et al., 2013; Lemanski et al., 2019). When faced with that limiting environment, the soil microbial community may attempt to compensate for functional constraints induced by an insufficient P supply by enhancing the expression and release of P-related hydrolases. From the perspective of biogeochemical mechanisms, both C and N can be continually replenished via ecological processes, such as nitrogen fixation and litter decomposition, whereas the main input of P depends on the weathering of parent materials and the release of mineral phosphorus after the breakdown of soil aggregates (Heuck et al., 2015). The conversion efficiency of P to available forms is relatively low, however.

This study mainly relies on enzyme activity ratios, such as BG + CBH/AP, to infer the scope of nutrient limitation in afforested sandy soils, but does not consider possible non-specific effects of environmental factors on enzyme activity. The pH of soil can influence the phosphatase activity component of microbial demand, particularly in alkaline soils where AP activity tends to be higher (Acosta-Martínez and Tabatabai, 2000). However, the sandy soil in our study area is always alkaline and its spatial variation is relatively small (Yu T. et al., 2024), suggesting that any pH effects would be consistent across the sampled shrub stands and cannot explain the systematic increase in AP with stand age. Thus, the observed increase in AP activity along the chronosequence, together with its parallel increase in C- and N-acquiring enzymes and strong correlations with soil C: P and N: P ratios, supports our current interpretation that this trend reflects a rising microbial P demand rather than non-specific pH artifacts (Moorhead et al., 2016). Future studies should nonetheless incorporate direct measurements of soil pH, moisture, temperature, and other physicochemical properties to further disentangle the relative contributions of abiotic and biotic drivers to extracellular enzyme stoichiometry in afforestated ecosystems.

Interestingly, despite the overall P limitation, the degree of P limitation decreased with stand age (Figure 5d). This trend may be explained by several non-mutually exclusive mechanisms. First, older stands accumulate more organic matter and microbial biomass, which may enhance the mineralization of organic P via increased phosphatase activity and microbial turnover. Second, the progressive development of the rhizosphere in older stands, including greater root exudation and expanded mycorrhizal associations, may improve P mobilization and acquisition efficiency (Smith and Read, 2008). Third, although soil C: P and N: P ratios increase, the absolute pool of available P may become more biologically accessible over time due to improved soil structure and aggregate stability. These possibilities warrant further investigation through targeted experiments.

In the present study, the type of nutrient limitation faced by soil microbes remained the same through the afforestation period, but this is not always the case for all ecosystems. For instance, Zhang et al. (2019) found a gradual shift from P to N limitation for soil microbes during afforestation with *Robinia pseudoacacia* planted on the Loess Plateau. Wang et al. (2023) noted that the accumulation of available P in soil during the later stage of soil development could alleviate P limitation in subtropical tree plantations, but the relative insufficiency of N usually induces chronic microbial N limitation. In our study, however, N limitation did not occur in stages of the afforestation chronosequence. This discrepancy is arguably due to the N-fixation ability of *C. korshinskii*, which forms symbiotic relationships with rhizobia to convert atmospheric N_2_ into bioavailable N, thereby providing a stable and continuous N source input to soil (Jia et al., 2022). As its stands aged, the soil STN increased at least six-fold (from 0.08 to 0.51 g·kg^−1^), while soil N: P increased at least five-fold (from 0.62 to 3.32; Table 1), confirming the continual accumulation of soil N across the afforestation period spanning seven decades. Other research has demonstrated that the litter of legume species has a lower C: N ratio and a faster decomposition rate, which hastens the turnover of N in the soil–microorganism system (Cleveland and Liptzin, 2007). So, even in the later stages of afforestation (50–70 years), by which time the shrub vegetation biomass had increased considerably along with greater competition for nutrients by soil microbes, the successional ecosystem could still ensure a sufficient N supply, thus effectively precluding the onset of N limitation in the Mu Us Desert. This finding agrees with that of Waring et al. (2014) for global tropical soils, where the presence of N-fixing plants exacerbates the transition from N to P limitation faced by microbes belowground.

We also found that soil microbial C limitation intensifies drastically in the 0–10-year interval of afforestation, but it generally stabilizes afterward as the shrub stands get older. This asymptotic trend is closely related to the temporal dynamics of soil C input. Soon after planting, the *C. korshinskii* shrubs were still small and mostly juveniles, whose shed leaves and root exudates inputted relatively little C; but as the stands developed and the plants got larger and attained reproductive maturity, the growth and metabolism of belowground microbes accelerated, and their C demand increased, intensifying their C limitation (Deng et al., 2013). The non-exacerbated C limitation beyond 10 years is partly attributable to accumulated fallen leaves and root biomass, which greatly amplified external C inputs (Ruiz-Jaén and Aide, 2005). Furthermore, rhizosphere development in older stands enhances root-derived C inputs and stimulates microbial processing of labile C compounds, thereby accelerating the formation of stable soil organic matter through microbial necromass accumulation and mineral-associated organic matter formation (Jia et al., 2022; Huang et al., 2025). On the other hand, the C sources accruing in the earlier stage of afforestation with *C. korshinskii* promote the growth of microbial biomass, along with necromass C and its metabolites, which may serve as vital sources of a stable soil C pool (Huang et al., 2025), thus potentially contributing to soil C’s replenishment. This dual buildup of plant and microbial C seems to effectively rectify the insufficient C supply. Therefore, in ecologically restoring the Mu Us Desert via afforestation with *C. korshinskii*, C limitation only intensified transiently, in the initial stage, after which it hardly changed.

To sum up, the microbial nutrient limitation dynamics during the *C. korshinskii* afforestation process in the Mu Us Desert present a unique, intriguing pattern. It is dominated by long-term P limitation, with no notable N limitation, yet accompanied by early C limitation that then eases and remains stable. The formation of this limitation pattern, as suggested by the multiple regression modeling, pinpoints EEA C: P in particular, and to a much lesser extent EEA C: N, as crucial indicators for predicting the scope of microbial nutrient limitation. How this limitation pattern arises is jointly determined by plant traits (e.g., N-fixation ability of *C. korshinskii*), the soil’s background attributes (e.g., slow conversion of P in arid zones), and chemical metric characteristics of the successional ecosystem. Among these indicators, EEA C: P showed the strongest association (Figure 6). This is likely because the C: P ratio of extracellular enzymes directly integrates the relative investment of microbes in carbon versus phosphorus acquisition, making it a sensitive indicator of stoichiometric imbalance between energy supply and a critically limited nutrient. Looking ahead, future management of sand-fixation shrub stands should utilize sensitive indicators, namely EEA C: P, for the early warning and dynamic assessment of the soil microbial functional state during afforestation. we suggest that future experimental studies, such as controlled P-addition trials, are needed to validate the inferred P limitation and to guide potential management interventions. Doing so would provide a sound scientific basis and technical support for the sustainable restoration of desert-like ecosystems in arid regions.

## 5 Conclusion

This study applied ecological stoichiometry theory, combined with the vector analysis modeling, to systematically explore the coordinated patterning of chemical stoichiometric characteristics of soil, microorganisms and extracellular enzymes in the *C. korshinskii* sand-fixing stands differing in age (planted 10, 30, 50 and 70 years ago), as well as the dynamic changes in their microbial nutrient limitations. Our results support the following main conclusions. Establishing sand-fixing vegetation stands using the *C. korshinskii* shrub alters the stoichiometric ratios of C, N, and P among soil, microorganisms and extracellular enzymes, driving an imbalance in soil element homeostasis. The relative C limitation of soil microbes initially increases after 10 years and then stabilizes. From 0 through 70 years of the afforestation chronosequence, soil microbes are always suggested to be P-limited but the degree of this limitation declines over time. The C: P ratio (and to some extent the C: N ratio) of extracellular enzymes is a closely associated indicator for predicting microbial nutrient limitations. The research findings provide a better scientific understanding of microbial nutrient regulation mechanisms in the artificial afforestation process of degraded desert-like ecosystems, and offer theoretical support for adaptive management in the ecological restoration of arid regions.

## Data Availability

The original contributions presented in the study are included in the article/supplementary material. Further inquiries can be directed to the corresponding author.

## References

[ref1] Acosta-Martínez V. Tabatabai M. A. (2000). Enzyme activities in a limed agricultural soil. Biol. Fertil. Soils 31, 85–91. doi: 10.1007/s003740050628

[ref2] Adamczyk B. Kilpeläinen P. Kitunen V. Smolander A. (2014). Potential activities of enzymes involved in N, C, P and S cycling in boreal forest soil under different tree species. Pedobiologia 57, 97–102. doi: 10.1016/j.pedobi.2013.12.003

[ref3] Arenberg M. R. Arai Y. (2019). Uncertainties in soil physicochemical factors controlling phosphorus mineralization and immobilization processes. Adv. Agron. 154, 153–200. doi: 10.1016/bs.agron.2018.11.005

[ref4] Armstrong R. A. Hilton A. C. (2010). “Stepwise Multiple Regression,” in Statistical Analysis in Microbiology: Statnotes, eds. Armstrong R. A. Hilton A. C. ().

[ref5] Bao S. D. (2000). Soil and Agricultural Chemistry Analysis. 3rd Edn Beijing, China: China Agriculture Press.

[ref6] Burns R. G. Deforest J. L. Marxsen J. Sinsabaugh R. L. Stromberger M. E. Wallenstein M. D. . (2013). Soil enzymes in a changing environment: current knowledge and future directions. Soil Biol. Biochem. 58, 216–234. doi: 10.1016/j.soilbio.2012.11.009

[ref7] Cleveland C. C. Liptzin D. (2007). C: N: P stoichiometry in soil: is there a “Redfield ratio” for the microbial biomass? Biogeochemistry 85, 235–252. doi: 10.1007/S10533-007-9132-0

[ref8] Cui Y. Moorhead D. L. Peng S. Sinsabaugh R. L. (2023). New insights into the patterns of ecoenzymatic stoichiometry in soil and sediment. Soil Biol. Biochem. 177:108910. doi: 10.1016/j.soilbio.2022.108910

[ref9] Deng L. Shangguan Z. P. Sweeney S. (2013). Changes in soil carbon and nitrogen following land abandonment of farmland on the loess plateau, China. PLoS One 8:e71923. doi: 10.1371/journal.pone.0071923 PMC3733848 23940793

[ref10] Guo X. Yang G. Wang C. G. Ren Y. Han X. Wang N. . (2025). Improvement effects of different shrub sand fixation plantations on vegetation and soil in the Kubuqi Desert. Front. Plant Sci. 16:1688154. doi: 10.3389/fpls.2025.1688154, 41358342 PMC12678344

[ref11] Heuck C. Weig A. Spohn M. (2015). Soil microbial biomass C: N: P stoichiometry and microbial use of organic phosphorus. Soil Biol. Biochem. 85, 119–129. doi: 10.1016/j.soilbio.2015.02.029

[ref12] Huang N. He H. Y. Fan R. Li X. Y. Zhao C. M. Li J. H. (2025). Planting of nitrogen-fixing shrubs promote soil carbon sequestration by increasing mineral-associated organic fraction. Geoderma 457:117282. doi: 10.1016/j.geoderma.2025.117282, 42574925

[ref13] Huang H. Tian D. Zhou L. Su H. Ma S. Feng Y. . (2022). Effects of afforestation on soil microbial diversity and enzyme activity: a meta-analysis. Geoderma 423:115961. doi: 10.1016/j.geoderma.2022.115961

[ref14] Jia B. Jia L. Zhang Y. Mou X. M. Li X. G. (2022). Leguminous *Caragana korshinskii* evidently enhances microbial necromass carbon accumulation in dryland soils. Catena 215:106342. doi: 10.1016/j.catena.2022.106342

[ref15] Jiao F. Wen Z. M. An S. S. Yuan Z. (2013). Successional changes in soil stoichiometry after land abandonment in loess plateau, China. Ecol. Eng. 58, 249–254. doi: 10.1016/j.ecoleng.2013.06.036

[ref16] Kaya A. Gkbulak F. Salam R. Erkan N. (2026). Long-term impacts of conifer afforestation on forest floor development and soil properties in herbaceous rangelands under semi-arid and sub-humid climates. Land Degrad. Dev. 37:2239. doi: 10.12911/22998993/185354

[ref17] Kunito T. Moro H. Mise K. Sawada K. Otsuka S. Nagaoka K. . (2024). Ecoenzymatic stoichiometry as a temporally integrated indicator of nutrient availability in soils. Soil Sci. Plant Nutr. 70, 246–269. doi: 10.1080/00380768.2024.2341669

[ref18] Lemanski K. Armbruster M. Bonkowski M. (2019). Linking soil microbial nutrient limitation to fertilizer regime and sugar beet yield. Plant Soil 441, 253–259. doi: 10.1007/s11104-019-04114-w

[ref19] Li Y. Han C. Sun S. Zhao C. (2021). Effects of tree species and soil enzyme activities on soil nutrients in dryland plantations. Forests 12:1153. doi: 10.21203/rs.3.rs-681222/v1

[ref20] Liang C. Schimel J. P. Jastrow J. D. (2017). The importance of anabolism in microbial control over soil carbon storage. Nat. Microbiol. 2:17105. doi: 10.1038/nmicrobiol.2017.105, 28741607

[ref21] Moorhead D. L. Rinkes Z. L. Sinsabaugh R. L. Weintraub M. N. (2013). Dynamic relationships between microbial biomass, respiration, inorganic nutrients and enzyme activities: informing enzyme-based decomposition models. Front. Microbiol. 4:233. doi: 10.3389/fmicb.2013.00223, 23964272 PMC3740267

[ref22] Moorhead D. L. Sinsabaugh R. L. Hill B. H. Weintraub M. N. (2016). Vector analysis of ecoenzyme activities reveal constraints on coupled C, N and P dynamics. Soil Biol. Biochem. 93, 1–7. doi: 10.1016/j.soilbio.2015.10.019

[ref23] Ruiz-Jaén M. C. Aide T. M. (2005). Vegetation structure, species diversity, and ecosystem processes as measures of restoration success. For. Ecol. Manag. 218, 159–173. doi: 10.1016/j.foreco.2005.07.008

[ref24] Saiya-Cork K. Sinsabaugh R. Zak D. (2002). The effects of long term nitrogen deposition on extracellular enzyme activity in an *Acer saccharum* forest soil. Soil Biol. Biochem. 34, 1309–1315. doi: 10.1016/S0038-0717(02)00074-3

[ref25] Schimel J. P. Weintraub M. N. (2003). The implications of exoenzyme activity on microbial carbon and nitrogen limitation in soil: a theoretical model. Soil Biol. Biochem. 35, 549–563. doi: 10.1016/S0038-0717(03)00015-4

[ref26] Shu M. Wang D. L. Wang K. Lian Z. Tang J. X. Han X. M. . (2018). Ecological stoichiometry characteristics of needle leaves-litter-soils in *Pinus sylvestris* var. Mongolica plantations with different forest ages. J. Soil Water Conserv. 32, 174–179. doi: 10.13870/j.cnki.stbcxb.2018.03.027

[ref27] Sinsabaugh R. L. Follstad Shah J. J. (2012). Ecoenzymatic stoichiometry and ecological theory. Annu. Rev. Ecol. Evol. Syst. 43, 313–343. doi: 10.1146/annurev-ecolsys-071112-124414

[ref28] Sinsabaugh R. L. Hill B. H. Follstad Shah J. J. (2009). Ecoenzymatic stoichiometry of microbial organic nutrient acquisition in soil and sediment. Nature 462, 795–798. doi: 10.1038/nature08632, 20010687

[ref29] Sinsabaugh R. L. Lauber C. L. Weintraub M. N. Ahmed B. Allison S. D. Crenshaw C. . (2008). Stoichiometry of soil enzyme activity at global scale. Ecol. Lett. 11, 1252–1264. doi: 10.1111/j.1461-0248.2008.01245.x, 18823393

[ref9001] Smith S. E. Read D. J. (2008). Mycorrhizal Symbiosis. 3rd Edn. Amsterdam: Academic Press.

[ref30] Tian H. Chen G. Zhang C. Melillo J. M. Hall C. A. S. (2010). Pattern and variation of C:N:P ratios in China's soils: a synthesis of observational data. Biogeochemistry 98, 139–151. doi: 10.1007/S10533-009-9382-0

[ref31] Wang J. Chen G. Ji S. Zhong Y. Zhao Q. He Q. . (2023). Close relationship between the gene abundance and activity of soil extracellular enzyme: evidence from a vegetation restoration chronosequence. Soil Biol. Biochem. 177:108929. doi: 10.1016/j.soilbio.2022.108929

[ref32] Wang C. Q. Jiao R. Z. (2022). Adaptive pathways of microorganisms to cope with the shift from P-to N-limitation in subtropical plantations. Front. Microbiol. 13:870667. doi: 10.3389/fmicb.2022.870667, 35572659 PMC9100944

[ref33] Waring B. G. Weintraub S. R. Sinsabaugh R. L. (2014). Ecoenzymatic stoichiometry of microbial nutrient acquisition in tropical soils. Biogeochemistry 117, 101–113. doi: 10.1007/s10533-013-9849-x

[ref34] Wu W. Chen G. Meng T. Li C. Feng H. Si B. . (2023). Effect of different vegetation restoration on soil properties in the semi-arid loess plateau of China. Catena 220:106630 (PartA): 106630. doi: 10.1016/j.catena.2022.106630, 42574925

[ref35] Wu J. S. Lin Q. M. Huang Q. Y. Xiao H. A. (2006). Measurement Method and Application of Soil Microbial Biomass. Beijing: China Meteorological Press, 54–84.

[ref36] Wu X. Z. Yan X. Wang B. Liu R.-T. An H. (2019). Effects of desertification on the C:N:P stoichiometry of soil, microbes, and extracellular enzymes in a desert grassland. Chin. J. Plant Ecol. 42, 1022–1032. doi: 10.17521/cjpe.2018.0121

[ref38] Xiao L. Min X. X. Liu G. B. Min X. Liu G. Li P. . (2023). Effect of plant–plant interactions and drought stress on the response of soil nutrient contents, enzyme activities and microbial metabolic limitations. Appl. Soil Ecol. 181:104666. doi: 10.1016/j.apsoil.2022.104666

[ref39] Xu Z. Liang W. Lei J. Wu Y. Wang Z. (2025). Monitoring and assessment of desertification reversal in ecologically fragile areas: a case study of the mu us Sandy land. J. Environ. Manag. 373:695. doi: 10.1016/j.jenvman.2024.123695, 39693988

[ref40] Yu T. Feng Q. Yin Y. Han T. Chen W. Zhu M. . (2024). Divergent response of upper layer soil organic and inorganic carbon to biotic and abiotic factors in afforestation by aerial seeding in desert, China. Catena 246:108383. doi: 10.1016/j.catena.2024.108383

[ref41] Yu S. Yang J. L. Norghauer J. M. Yang J. Yang B. Zhang H. . (2024). Soil carbon and nitrogen stocks and their influencing factors in different-aged stands of sand-fixing *Caragana korshinskii* in the mu Us Desert of Northwest China. Forests 15:1018. doi: 10.3390/f15061018

[ref42] Zhan X. L. Han L. (2015). Physicochemical property of aeolian sandy soil in Baijitan in Ningxia. Chin. Agric. Sci. Bull. 31:186. doi: 10.11924/j.issn.1000-6850.2014-1781

[ref43] Zhang X. Li W. Zhong Z. Zhang Q. Wang X. Han X. . (2020). Response of soil microbial community to C:N:P stoichiometry along a *Caragana korshinskii* restoration gradient on the loess plateau, China. Forests 11:823. doi: 10.3390/f11080823

[ref44] Zhang W. Liu W. C. Xu M. P. Liu W. Xu M. Deng J. . (2019). Response of forest growth to C:N:P stoichiometry in plants and soils during *Robinia pseudoacacia* afforestation on the loess plateau, China. Geoderma 337, 280–289. doi: 10.1016/j.geoderma.2018.09.042

[ref45] Zhang K. Su Y. Z. Wang T. Liu T. N. (2016). Soil stoichiometry characteristics of Haloxylon ammodendron with different plantation age in the desert-oasis ecotone, North China. Acta Ecol. Sin. 36, 3235–3243. doi: 10.5846/stxb201506301362

[ref46] Zhao P. B. He Y. Huang Y. Y. Zhao X. J. Chen L. Liu W. S. . (2021). Spatial heterogeneity of soil nutrients under different stand-fixing shrubs in southern mu us sandy land, China. Acta Agrestia Sin. 29, 2040–2048. doi: 10.1016/S0167-8809(01)00217-1

